# Correction: Li et al. Whole-Genome Sequence Analysis of *Flammulina filiformis* and Functional Validation of *Gad*, a Key Gene for γ-Aminobutyric Acid Synthesis. *J. Fungi* 2024, *10*, 862

**DOI:** 10.3390/jof11080605

**Published:** 2025-08-20

**Authors:** Wenyun Li, Junjun Shang, Dapeng Bao, Jianing Wan, Chenli Zhou, Zhan Feng, Hewen Li, Youran Shao, Yingying Wu

**Affiliations:** 1 College of Food Sciences & Technology, Shanghai Ocean University, Shanghai 201306, China; liwenyun1209@126.com (W.L.); shangjunjun@saas.sh.cn (J.S.); baodapeng@saas.sh.cn (D.B.); 2 National Engineering Research Center of Edible Fungi, Key Laboratory of Applied Mycological Resources and Utilization of Ministry of Agriculture, Institute of Edible Fungi, Shanghai Academy of Agricultural Sciences, Shanghai 201403, China; wanjianing@163.com (J.W.); zhouchenli@saas.sh.cn (C.Z.); 3 Jiangsu Chinagreen Biological Technology Co., Ltd., Siyang 223700, China; fz@chinagreenbio.com (Z.F.); hltk002@chinagreenbio.com (H.L.)

In the original publication [1], there was a mistake in Table 3: the units of three rows were incorrectly labeled as kb instead of bp. The corrected table appears below. The authors state that the scientific conclusions are unaffected. This correction was approved by the Academic Editor. The original publication has also been updated.

**Table 3:** Summarized genome information for *Fv-HL23-1*.

Assembly Statistics	Scaffolds
Genome size (Mb)	40.96
Total sequence number	140
Total sequenced length (bp)	35,904,424
Maximum sequence length (bp)	2,467,745
Minimum sequence length (bp)	5510
GC (%)	49.62
N20 length (bp)	1,658,848
N50 length (bp)	917,125
N90 length (bp)	141,141
Total gene length (bp)	23,522,018
Gene Percentage of Genome (%)	65.51
Total number of genes	14,256
Average gene length (bp)	1649.9
Total exon length (bp)	19,949,251
Exon percentage of genome (%)	55.56
Average Exon Length (bp)	250.7
Total coding sequence (CDS) length (bp)	19,949,251
Average CDS length (bp)	1399.3
CDS percentage of genome (%)	55.56
Average intron length (bp)	54.7
Sequencing platform	PacBio CLR, Illumina

## References

[1] Li W., Shang J., Bao D., Wan J., Zhou C., Feng Z., Li H., Shao Y., Wu Y. (2024). Whole-Genome Sequence Analysis of *Flammulina filiformis* and Functional Validation of *Gad*, a Key Gene for γ-Aminobutyric Acid Synthesis. J. Fungi.

