# Supplementary material for: Whole-Genome Sequence Analysis of Flammulina filiformis and Functional Validation of Gad, a Key Gene for γ-Aminobutyric Acid Synthesis
Source: J Fungi (Basel). 2024 Dec 12;10(12):862. doi: 10.3390/jof10120862 (PMC11678480; doi:10.3390/jof10120862)
Supplement: Supplementary file 1 [file jof-10-00862-s001.zip › jof-3234590-supplement material.pdf]

Supplementary Material

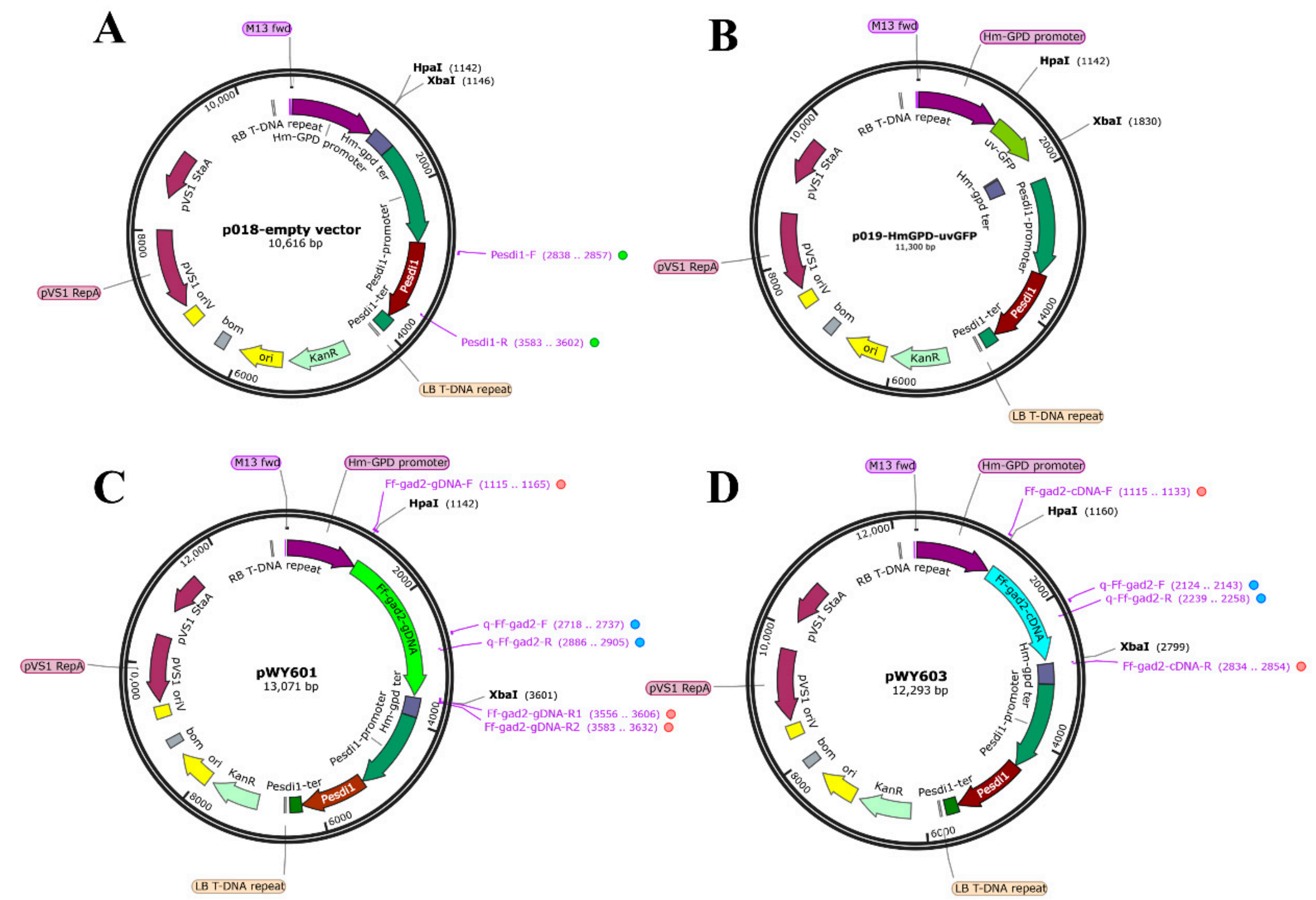

Figure S1. Summary of plasmid profiles used in this study. (A) p018-empty vector plasmid; (B) p019-HmGPD-uvGFP plasmid; (C) pWY601 plasmid; (D) pWY603 plasmid.

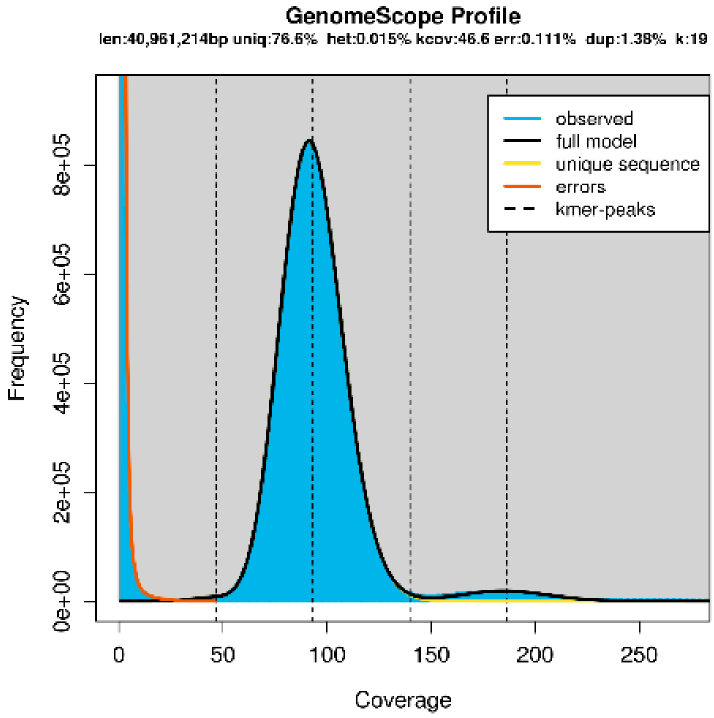

Figure S2. K-mer depth distribution of *Fv-HL23-1* genome (K=19).

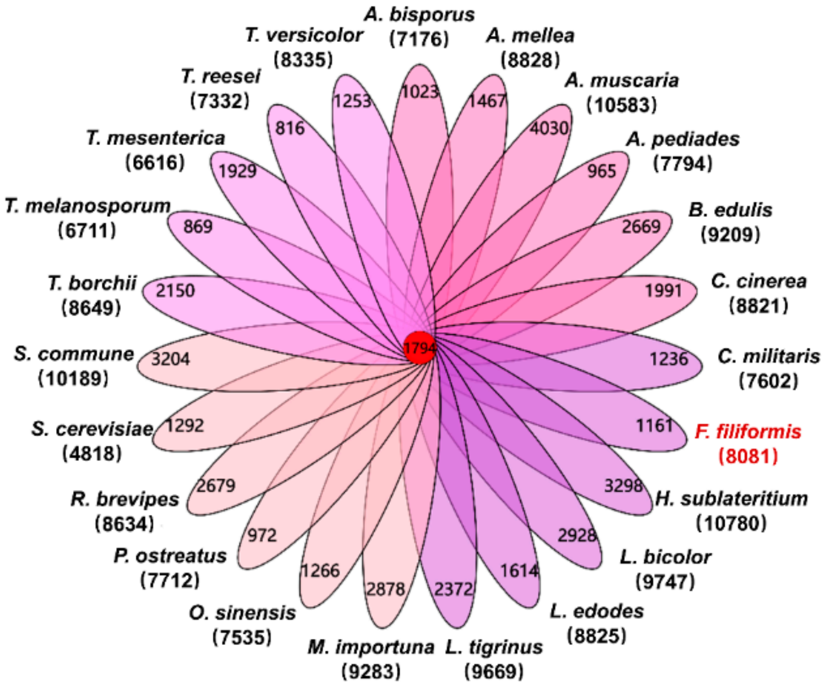

Figure S3. Venn diagram of orthologous gene families of 23 fungi. Each circle represents the total number of genes in a species, and numerical intersections represent the number of gene families shared by multiple species. The total number of genes contained in gene families shared by multiple species is represented in parentheses.

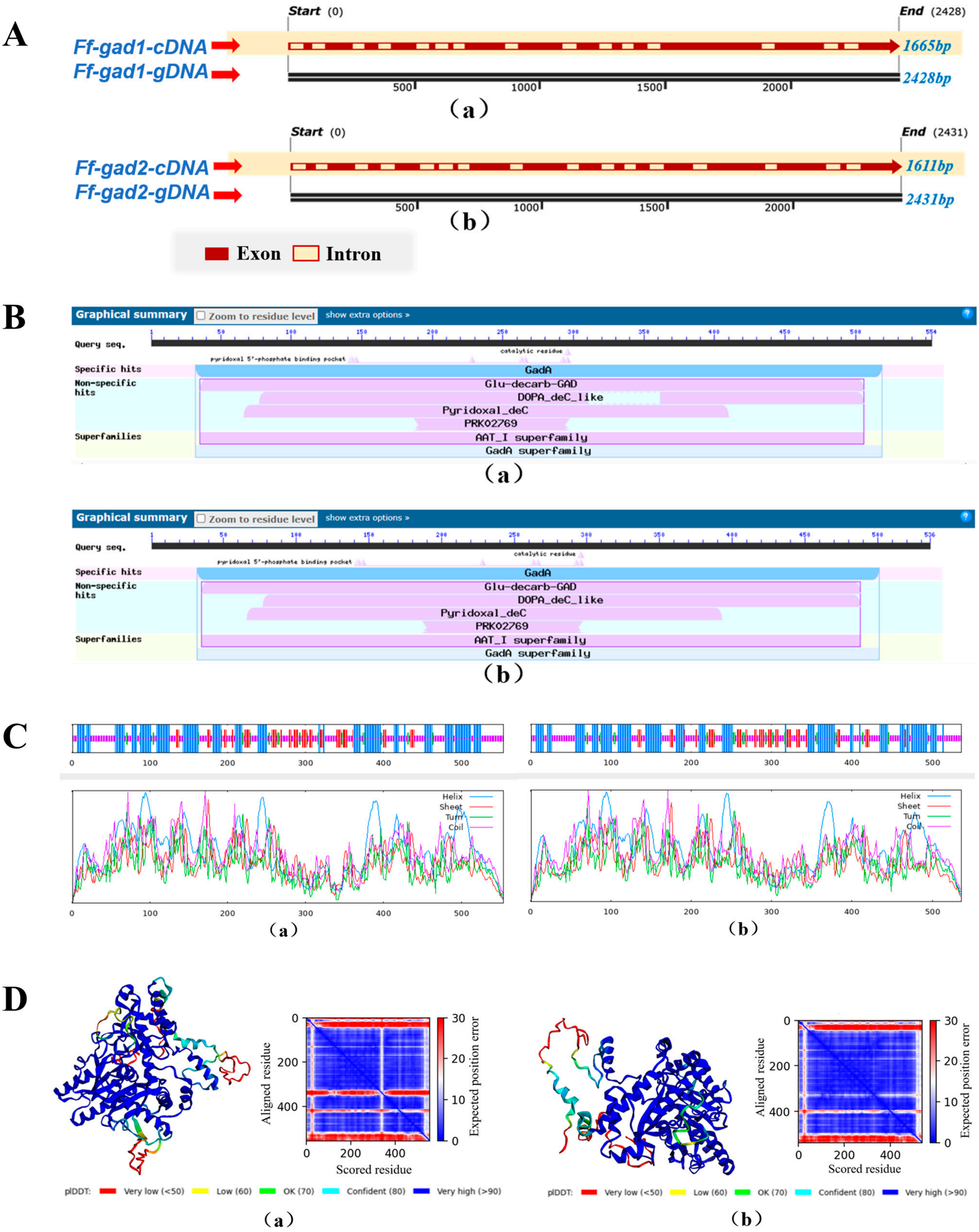

Figure S4. Bioinformatics analysis of Ff-GAD proteins. (A) The exon and intron distribution of the extracted *Ff-gad* gene using homologous comparison; (B) Prediction of Ff-GAD conserved domain; (C) Prediction of amino acid secondary structure of Ff-GAD; (D) AlphaFold2 prediction of amino acid tertiary structure and structure confidence map for Ff-GAD, where (a) is Ff-GAD1 and (b) is Ff-GAD2.

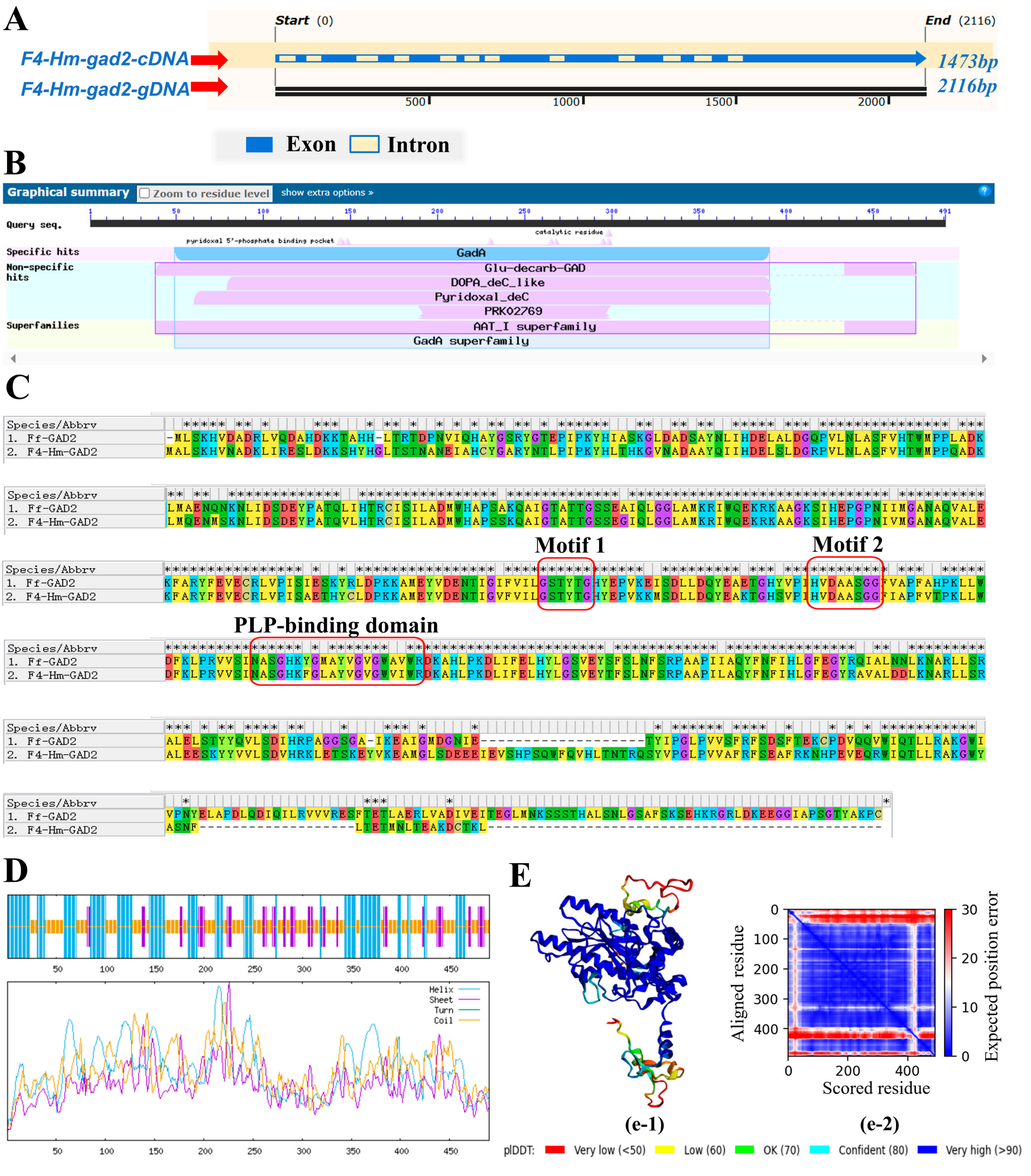

Figure S5. Bioinformatics analysis of F4-Hm-GAD proteins. (A) The exon and intron distribution of the extracted *F4-Hm-gad* gene using homologous comparison; (B) Prediction of F4-Hm-GAD conserved domain; (C) The results of multiple alignment of GAD sequences derived from *F. filiformis* and *H. marmoreus*; (D) Prediction of amino acid secondary structure of F4-Hm-GAD; (E) AlphaFold2 prediction of amino acid tertiary structure(e-1) and structure confidence map(e-2) for F4-Hm-GAD.

Table S1. List of genomic information for 22 fungal species.

Phylum	Name	Submitted GenBank assembly	Strain	Genome size/Mb	Number of contigs	GC percent/%	Date
Basidiomycota	*Lentinula edodes*	GCA_021015755.1	*Le(Bin) 0899 ss11*	45.6	128	46	Nov 30, 2021
Basidiomycota	*Lentinus tigrinus*	GCA_003813185.1	*ALCF2SS1-7*	39.5	207	56	Nov 21, 2018
Basidiomycota	*Agaricus bisporus*	GCA_000300575.1	*H97*	30.2	29	46.5	Nov 30, 2012
Basidiomycota	*Pleurotus ostreatus*	GCA_014466165.1	*PC9*	34.9	16	51	Sep 3, 2020
Basidiomycota	*Laccaria bicolor*	GCA_000143565.1	*S238N-H82*	64.9	4401	47	Jan 11, 2008
Basidiomycota	*Boletus edulis*	GCA_015179015.1	*BED1*	66.5	593	50.5	Nov 2, 2020
Basidiomycota	*Armillaria mellea*	GCA_030407055.1	*ELDO17*	70.9	474	48	Jul 5, 2023
Basidiomycota	*Russula brevipes*	GCA_022496215.1	*BPL707*	48.5	1320	53	Mar 7, 2022
Basidiomycota	*Agrocybe pediades*	GCA_013053245.1	*CBS 102.39*	45.1	70	48	May 12, 2020
Basidiomycota	*Tremella mesenterica*	GCA_000271645.1	*Fries*	28.6	484	46.5	Jun 22, 2012
Basidiomycota	*Trametes versicolor*	GCA_000271585.1	*FP-101664 SS1*	44.8	977	57.5	Jun 22, 2012
Basidiomycota	*Schizophyllum commune*	GCA_000143185.2	*H4-8*	38.7	72	57.5	May 17, 2022
Basidiomycota	*Coprinopsis cinerea*	GCA_000182895.1	*okayama7#130*	36.2	67	51.5	Jun 16, 2010
Basidiomycota	*Hypholoma sublateritium*	GCA_000827495.1	*FD-334 SS-4*	48	1342	51	Mar 2, 2015
Basidiomycota	*Amanita muscaria*	GCA_000827485.1	*Koide BX008*	40.7	3814	47.5	Jan 27, 2015
Ascomycota	*Morchella importuna*	GCA_003444635.2	*M04M26*	50.9	109	47.5	Sep 4, 2018
Ascomycota	*Cordyceps militaris*	GCA_000225605.1	*CM01*	32.3	597	51.5	Sep 12, 2011
Ascomycota	*Ophiocordyceps sinensis*	GCA_012934285.1	*IOZ07*	110.9	23	45	Apr 30, 2020
Ascomycota	*Tuber melanosporum*	GCA_000151645.1	*Mel28*	124.9	4440	45	May 3, 2010
Ascomycota	*Tuber borchii*	GCA_003070745.1	*Tbo3840*	97.2	969	46.5	Apr 24, 2018
Ascomycota	*Saccharomyces cerevisiae*	GCA_000146045.2	*S288C*	12.1	16	38.5	Dec 17, 2014
Ascomycota	*Trichoderma reesei*	GCA_000167675.2	*QM6a*	33.4	128	53	Jul 18, 2011

The data and related information in the table are from the official website of National Center for Biotechnology Information (NCBI).

Table S2. Statistical results for gene families of 23 fungi.

Species	Total genes	Unclustered genes	Unique paralogs	Single-copy orthologs	Multiple-copy orthologs	Other orthologs
M. importuna	11642	2698	778	1585	506	6075
C. militaris	9651	1168	210	1587	619	6067
O. sinensis	9961	1082	499	1447	862	6071
T. melanosporum	7420	854	55	1585	491	4435
T. borchii	12345	1857	1532	1541	657	6758
S. cerevisiae	6014	1156	480	1473	768	2137
T. reesei	9111	766	129	1600	560	6056
L. edodes	14078	1202	1638	1445	1069	8724
F. filiformis	14256	713	2404	1486	897	8756
L. tigrinus	15372	1980	1409	1526	892	9565
A. bisporus	10448	869	1043	1570	612	6354
P. ostreatus	11717	660	1195	1535	761	7566
L. bicolor	18213	2078	3891	1423	1087	9734
B. edulis	18718	1715	4572	1380	1277	9774
A. mellea	15646	1086	1477	1468	1073	10542
R. brevipes	13996	2159	2423	1501	843	7070
A. pediades	12919	711	1334	1540	796	8538
T. mesenterica	8291	1728	1230	1654	338	3341
T. versicolor	14292	936	1300	1552	833	9671
S. commune	16193	2636	2599	1516	866	8576
C. cinerea	13356	1623	1577	1542	709	7905
H. sublateritium	17771	2732	2230	1450	993	10366
A. muscaria	18093	3431	2661	1482	984	9535

Table S3. Data on basic features of proteins encoded by FfCYPs of *Fv-HL23-1*.

Name	Protein length(aa)	Molecular weight（kDa）	Name	Protein length(aa)	Molecular weight（kDa）
scaffold1.t136	551	60.72	scaffold29.t81	534	59.12
scaffold1.t276	481	53.61	scaffold3.t67	538	61.10
scaffold1.t346	379	41.95	scaffold3.t93	522	57.64
scaffold1.t347	155	17.23	scaffold3.t216	521	58.26
scaffold1.t348	499	55.94	scaffold3.t391	534	59.59
scaffold1.t848	572	63.96	scaffold3.t405	543	60.59
scaffold1.t915	521	58.84	scaffold3.t565	556	62.81
scaffold1.t1046	489	54.22	scaffold3.t722	530	60.49
scaffold10.t59	507	57.79	scaffold3.t743	538	60.08
scaffold10.t426	540	59.96	scaffold30.t26	815	91.25
scaffold104.t2	516	57.80	scaffold31.t90	594	67.47
scaffold11.t102	506	56.01	scaffold31.t91	540	60.99
scaffold11.t117	526	58.54	scaffold31.t101	517	57.95
scaffold11.t370	516	57.61	scaffold33.t10	476	53.09
scaffold11.t400	501	56.58	scaffold34.t18	548	61.88
scaffold12.t157	423	47.81	scaffold35.t88	530	58.57
scaffold12.t158	494	55.38	scaffold35.t89	535	59.64
scaffold12.t160	461	51.61	scaffold35.t90	537	59.51
scaffold12.t307	482	54.24	scaffold37.t21	590	67.06
scaffold13.t35	603	68.82	scaffold38.t90	537	60.00

scaffold15.t83	374	42.02	scaffold4.t127	509	57.32
scaffold15.t156	484	54.05	scaffold4.t339	361	40.76
scaffold15.t208	567	63.67	scaffold41.t68	534	59.67
scaffold16.t165	546	62.49	scaffold42.t66	632	72.39
scaffold17.t69	423	48.31	scaffold45.t48	523	58.60
scaffold18.t145	522	58.74	scaffold47.t5	527	59.61
scaffold18.t148	524	58.48	scaffold5.t196	516	57.57
scaffold18.t186	360	39.10	scaffold5.t264	311	35.18
scaffold2.t316	499	56.33	scaffold5.t450	128	14.11
scaffold2.t513	514	58.74	scaffold51.t26	491	55.76
scaffold2.t564	534	59.46	scaffold52.t22	528	59.89
scaffold2.t703	501	55.75	scaffold52.t36	543	60.95
scaffold20.t23	538	60.88	scaffold52.t56	498	55.94
scaffold20.t35	539	59.23	scaffold6.t140	482	54.92
scaffold20.t51	538	59.61	scaffold6.t375	174	19.03
scaffold20.t52	536	59.11	scaffold63.t11	525	59.74
scaffold23.t115	442	49.70	scaffold63.t12	520	59.12
scaffold24.t85	536	59.98	scaffold63.t13	507	57.56
scaffold24.t115	494	55.18	scaffold63.t14	467	52.47
scaffold24.t122	583	66.59	scaffold63.t15	500	56.51
scaffold24.t126	551	61.22	scaffold63.t4	508	57.48
scaffold25.t16	548	62.48	scaffold63.t5	505	57.19
scaffold25.t34	518	58.55	scaffold63.t6	403	45.26
scaffold25.t36	455	51.20	scaffold7.t254	383	42.46
scaffold25.t74	885	100.32	scaffold72.t19	598	69.02
scaffold26.t10	554	58.94	scaffold8.t190	595	67.95
scaffold29.t22	521	58.11	scaffold8.t249	517	58.34
scaffold29.t23	489	54.30	scaffold8.t254	461	52.10
scaffold29.t29	137	15.10	scaffold84.t7	500	56.39
scaffold29.t33	167	18.58	scaffold84.t8	503	57.14
scaffold29.t34	129	14.48	scaffold84.t9	502	57.03
scaffold29.t51	506	57.28	scaffold84.t17	81	9.16
scaffold29.t59	517	57.92	scaffold9.t338	500	55.87
scaffold29.t79	534	59.07	scaffold9.t472	685	75.76

Table S4. Statistics of the T-DNA insertion sites in *H. marmoreus*.

Transformant	Position of deletion	Deletion length (bp)	DNA insertional position in plasmid	Insertion length (bp)	Orientation
pWY601-1	Scaffold2:1480531-1480637	107	pWY601:12825-13071,1-6571	6818	reverse T-DNA inserted to reference genome
pWY601-2	Scaffold35:304712-304730	19	pWY601:571-7875	7305	forward T-DNA inserted to reference genome
pWY601-3	Scaffold17:467639-467661	23	pWY601:13064-13071,1-6949	6957	forward T-DNA inserted to reference genome
pWY603-1	Scaffold35:253678-253750	72	pWY603:918-5793	4875	forward T-DNA inserted to reference genome
pWY603-2	Scaffold20:321429-321449	20	pWY603:12047-12293,1-5788	6035	forward T-DNA inserted to reference genome
pWY603-3	Scaffold24:548634-548787	154	pWY603:951-6037	5086	forward T-DNA inserted to reference genome
